# How do Norwegian adolescents experience the role of social media in relation to mental health and well-being: a qualitative study

**DOI:** 10.1186/s40359-021-00582-x

**Published:** 2021-05-13

**Authors:** Gunnhild Johnsen Hjetland, Viktor Schønning, Randi Træland Hella, Marius Veseth, Jens Christoffer Skogen

**Affiliations:** 1 Department of Health Promotion, Norwegian Institute of Public Health, Bergen, Norway; 2 Department of Work, Section for Children, Families and Disabled, Social Services and Housing, City of Bergen, Norway; 3 Department of Clinical Psychology, University of Bergen, Bergen, Norway; 4 Department of Public Health, Faculty of Health Sciences, University of Stavanger, Stavanger, Norway; 5 Alcohol and Drug Research Western Norway, Stavanger University Hospital, Stavanger, Norway

## Abstract

**Background:**

During the last decade, social media has permeated most parts of society. Adolescents are particularly active users of social media, and their use has been suggested as a contributing factor to mental health issues in this group. Quantitative studies have found associations between the frequency and/or duration of social media use and more mental health issues. However, most studies are cross-sectional and the identified associations are weak and of questionable practical significance. The aim of this study was to investigate adolescents’ lived experiences of using social media, focusing on both negative and positive aspects, using a qualitative approach. Qualitative research enables in-depth explorations of the experiences of individuals, nuance quantitative findings, and offer the perspective of adolescents into policies regarding social media use.

**Methods:**

Experiences and perspectives of 27 adolescents from two senior high schools in Norway were gathered using focus group interviews (mean age 16.8, range 15–18). The data were analysed using thematic analysis.

**Results:**

According to the participants, social media use catalyse changes to how people communicate and relate to each other (theme 1). Some changes were positive, in terms of facilitating social connectedness, while others were negative, such as people behaving worse than in face-to-face interactions. Further, social media use affected the participants at the individual level (theme 2); positively in terms of a sense of belonging and social support, and negatively in terms of perceived stress, body pressure, and displacing other activities such as sleep and homework. The motivations for using, or not using, social media were multifaceted (theme 3), reflecting the positive and negative aspects of social media in terms of interpersonal and personal consequences.

**Conclusion:**

Social media was described as an important part of the participants’ everyday life, with both a positive and negative impact on their mental health and well-being. Considering the compelling nature of social media and adolescents’ relatively limited self-regulatory capacities, efforts to modify social media use should avoid relying on self-regulation, while also recognizing the importance of social media as an arena for peer interaction.

**Supplementary Information:**

The online version contains supplementary material available at 10.1186/s40359-021-00582-x.

## Introduction

During the last decade, social media has become embedded in most parts of society. Using the definition by Kietzmann and colleagues [1, p. 1], social media are interactive platforms created by means of mobile and web-based technologies where individuals and communities share, discuss, co-create, and modify user-generated content. Social media use is particularly ubiquitous among adolescents and nearly half of US adolescent report using social media ‘almost constantly’ 2]. Among Norwegian adolescents, 47% spend at least two hours on social media every day [3], and well over 90% are on one or more social media platform [4].

During the last decade, increases in mental health problems such as anxiety and depression, and threats to mental health such as loneliness, have been reported among adolescents [5–9]. The widespread use of social media has been pointed to as a potential contributing cause [10], for example due to reduced face-to-face interactions [11], cyberbullying [12], and increased appearance-related social comparison [13].

As a relatively new phenomenon, the relationship between social media use and mental health has, however, not been extensively studied, and the quality of published studies are in general low according to a recent review [10]. The majority of studies have looked at quantitative measures of time spent on social media and the frequency of use in relation to different mental health outcomes and to a lesser extent specific behaviours on social media [14]. Studies using such measures of social media use have found that more time spent on social media is associated with symptoms of depression and anxiety [15–17], conduct problems, and episodic heavy drinking [15]. The identified associations are, however, small [18, 19] and with questionable practical consequences [9]. The underlying mechanisms in the observed relationship between the time spent on social media and mental health and well-being are likely to be multi-faceted [20]. Researchers have therefore called for more qualitative studies in order to identify potential mechanisms involved in the effects of social media on mental health [21].

Qualitative research enables in-depth explorations of the experiences of individuals [22, 23], and investigating the perspectives of adolescents using such approaches can nuance and make sense of the divergent quantitative findings and provide adolescents’ own perspective into policies regarding social media use. To date, a few qualitative studies of the role of social media in mental health have been conducted [24–27]. One study reported that the participants perceived social media as causing ill health [26]. In contrast, three studies demonstrated a duality in relation to social media, where participants saw social media use as affecting their mental health in both positive and negative ways [24, 25, 27]. Weinstein [25] reported how the same activities on social media could cause both positive and negative affective experiences. For example, interacting with peers could cause both feelings of closeness and feelings of isolation, and browsing social media could cause both admiration and envy. Throuvala et al. [24] explored motivational mechanisms behind social media use, and highlighted a need for control as a central motive driving social media omnipresence. Collectively, the studies highlighted the importance social media carries to adolescents in terms of communication with friends and family [24, 25], and that adolescents felt that this aspect of social media could protect their mental health [27].

The aim of this study was to elaborate further on the different roles of social media use in relation to mental health, in the perspective of Norwegian adolescents. We aimed to explore adolescents’ lived experiences of using social media, with a specific focus on how they view social media as facilitating and/or hindering their mental health. This knowledge can be used as a stepping stone on the way to develop guidelines for healthy social media use.

## Methods

The present study was an exploratory study using a focus group interview methodology [28–30]. The data were generated in the context of a larger project on social media and mental health, where the goal of the interviews was to generate questionnaire items for a large-scale longitudinal survey (see for instance [31]), and also to inform the development of a health promoting intervention programme involving social media. This paper reports on the results from the qualitative analysis of the focus group interviews.

### Study participants and setting

Respondents were recruited from two senior high schools in Norway. Five groups from two different schools were recruited, where one school was located in the city centre while the other was located in a rural area. Both schools offered general studies and different vocational education training programs. One of the schools had an e-sport program. The number of focus groups was set a priori, as aims to reach saturation was deemed non-relevant due to the highly exploratory nature of the study [32]. As most adolescents use social media, we anticipated that each participant held in-depth information about the topic of interest, and thus had high ‘information power’ [33]. Therefore, we expected that five groups would be sufficient to provide a rich and comprehensive insight into the lived experiences of the adolescents in the current context [33]. Twenty-seven youth from 15 to 18 years old (mean age 16.8) participated, and each group had 5–6 participants (Table 1). None of the participants withdrew from the study during or after the interview session.

**Table 1:** Simple demographic information about each participant

Participant ID	Focus group number	Age category	Gender
FG1-M1	1	17–18	Male
FG1-M2	1	17–18	Male
FG1-M3	1	17–18	Male
FG1-F1	1	17–18	Female
FG1-F2	1	17–18	Female
FG2-F1	2	17–18	Female
FG2-F1	2	15–16	Female
FG2-F2	2	17–18	Female
FG2-F3	2	15–16	Female
FG2-F4	2	17–18	Female
FG3-M1	3	15–16	Male
FG3-M2	3	15–16	Male
FG3-M3	3	17–18	Male
FG3-M4	3	15–16	Male
FG3-M5	3	17–18	Male
FG3-M6	3	17–18	Male
FG4-F1	4	17–18	Female
FG4-F2	4	17–18	Female
FG4-F3	4	15–16	Female
FG4-F4	4	17–18	Female
FG4-F5	4	17–18	Female
FG4-F6	4	15–16	Female
FG5-M1	5	17–18	Male
FG5-M2	5	15–16	Male
FG5-M3	5	15–16	Male
FG5-M4	5	17–18	Male
FG5-M5	5	17–18	Male

FG, focus group; M, male; F, female

A contact person at each school recruited participants to the focus groups in collaboration with the research group. The contact person knew the pupils and could thus recruit participants who would willingly participate in a group discussion and ensure a good group dynamic. Gender was regarded a dimension that could affect group dynamics and a central prerequisite for optimal homogeneity (gender as a break characteristic). Thus, two groups consisted of females only and two of males only, while one group included both males and females. The mixed gender group consisted of pupils attending the student council. One of the all-male groups included some pupils attending an e-sport educational program.

### Data collection

Prior to conducting the interviews, the researchers had a meeting with the participants at each school to talk about the project and to build initial rapport with the groups. Subsequently, the focus groups met once, for approximately 90 min, with a ten minute break halfway through. The interviews were completed at each school, using rooms located in the administrative parts of the buildings. The rooms had no windows or open sections leading into other indoor spaces, ensuring that the discussions among the participants could not be overheard or observed by others.

The interview guide was developed by the research group in collaboration with four Master-level psychologist scientist-practitioner students. The interview guide consisted of two main questions, each followed by a set of follow-up prompts that the moderator could use if necessary, as well as one opening question and two closing questions. The main questions were: (1) “In what way is social media a positive factor in your lives?” and (2) “In what way is social media a negative factor in your lives?” The interview guide was based on a template adapted on the background of preliminary results from a scoping review [34], as well as additional searches performed by the psychologist students. The complete guide was piloted on a group of adolescents (16–18 years), which led to some additional changes to the final interview guide. For example, the wording of some of the questions was altered, and more examples were included in the introductory text. The interview with the mixed gender group was completed about two weeks prior to the other four interviews to allow for any adjustment of the interview guide. However, the interview guide worked well and no adjustments were deemed necessary. The interview guide is available as Additional file 1.

The project coordinator (RTH) and one researcher (GJH) facilitated the interviews, alternately acting as the moderator and the secretary for each interview. The interviews were audiotaped and then transcribed verbatim. To ease the transcription of the interviews, the secretary wrote down the first few words of each sentence and who said it. In addition, the secretary took notes about aspects of the interview that are difficult to capture on tape, such as the non-verbal communication or the social climate of the group [28].

### Data analysis

We used reflexive thematic analysis (TA) to explore participants’ experiences [35, 36]. Following the six steps recommended by Braun and Clarke [35], we proceeded as follows:

1. Two researchers (GJH and RTH) read and re-read the material, drafting ideas for potential themes.
2. Subsequently, the researchers (GJH and RTH) independently identified relevant text and defined codes. After coding each interview, codes were compared and discussed, developing a rich reading of the data and generating meaningful codes. A third researcher (JCS) read all the material and participated in discussions about the generated codes.
3. GJH, RTH, and JCS sorted the codes into tentative themes. A fourth researcher (VS) provided an external perspective on the themes, following which all codes were collated under these themes.
4. GJH reviewed the themes against the coded text segments and against the transcribed interviews in full and discussed the themes in face-to-face and online meetings with JCS, RTH, VS, and MV.
5. When a consensus about the themes was reached, the themes were defined and given final names.

Reflexive TA was used to define patterns of meaning across the interviews. The coding process was guided by the overarching research questions in that we coded material that was relevant for mental health, well-being, and social media. The qualitative analysis included five meetings between the coders and four meetings with the whole group.

### Ethical considerations

Participation in the focus groups was voluntary. All participants gave their informed consent after being informed that they could withdraw at any time, and that they could choose how actively they wanted to participate in the discussion. Care was taken during the interviews to treat their experiences with dignity and respect, and we could see no indications that participants felt pressured to participate. However, the participants were recruited in a school setting, and the teacher–pupil relationship may have influenced willingness to participate.

When the interviews were transcribed, all participants’ names were replaced with fictitious names. All other identifiable information such as user names on social media or which school they attended, were replaced or omitted. The transcribed interviews were only available to those directly involved in transcription, coding, and analysis of the material. The audio files were permanently deleted following analysis.

## Results

The participants’ experiences with social media use and mental health and well-being converged under three main themes:

1. Interpersonal consequences of social media
2. Personal consequences of social media
3. Motivations affecting social media use

In relation to the main themes, several subthemes were developed (Table 2). Overall, social media was an important part of the participants’ everyday life, with both a positive and negative impact on their mental health and well-being. It became clear that the level of engagement with social media varied across participants; many used social media frequently and overtly expressed several positive and negative sides of social media use, while some had a more detached relationship to social media and did not see it as exerting much influence on them.

**Table 2:** An overview of the frequency of each theme/subtheme across the interviews

Theme/subtheme	Number of interviews the theme/subtheme occurred in
1. Interpersonal consequences of social media	5
1.1 Expanding the social world	5
1.2 Different rules apply	5
1.3 People behave worse on social media	5
2. Personal consequences of social media use	5
2.1 There are pros and cons of being connected	5
2.2 Social hierarchies are on display	4
2.3 Upward social comparison	4
2.4 The visibility and permanency of content	4
2.5 Use on the expense of other things	5
3. Motivations affecting use	5
3.1 An unmissable social arena	5
3.2 Self-presentation and impression management	5
3.3 From fun to addiction	5
3.4 A way to dodge what is difficult	5
3.5 Awareness and regulation of own use	5

An overview of the commonality of each theme and subtheme can be found in Table 2. As can be seen from the table, all three main themes and the majority of the subthemes were present in all focus group interviews, and the themes thus had broad coverage.

### Interpersonal consequences of social media

The participants discussed several ways in which social media affected communication and interaction, both positively and negatively. Three subthemes were generated: i) expanding the social world, ii) different rules apply, and iii) people behave worse on social media.

#### Expanding the social world

The participants said that social media facilitated their social lives by allowing them to communicate easier, maintain friendships, and also make new friends. Social networks were visible, and thus “everyone knew everyone” and going from that to being friends was easy: “Yes, I feel that it is much easier to get to know people. It’s like, you see that the people you are following are following someone, and then, like, everyone sort of knows each other” (Participant ID: FG3-M6). Further, social media facilitated group cohesion, for example within their class at school, by allowing multiple people to communicate continuously. One participant described how social media made him notice or pay more attention to his classmates. In a way, peoples’ social media profiles made them turn up on each other’s “social radar”.

This boost in social exchanges also had some downsides. Firstly, the participants talked about how many friendships on social media were superficial, and that they preferred face-to-face interactions. Some pruned their online network, deleting people that they did not see as real friends. One participant exemplified this side of social media:

I feel that even though [social media] enables more contact with friends and give me more friends, I also feel that, really, I have lost many friends. Because I feel that many of the friendships I have aren’t real friendships (FG1-F2).

Second, social media made them accessible to a wider range of people, including strangers. Unwanted attention on social media was discussed in four of the interviews, where the participants described receiving messages and group chat invitations from strangers. They were not particularly bothered by this, however, receiving sexualized content made them uncomfortable: “Nudes… people send you, like… A picture of their penis… I did not need to see that, I did not want to see that” (FG3-M5).

#### Different rules apply

Some of the participants described that a different set of communicative rules applied to social media, where the threshold for making contact was lower than in real life. Online communication was described as easy, informal, and less intrusive, compared to getting to know people outside social media:

I feel that, I don’t how it was before, but that it is easier to get to know people. There are, like, different rules on social media than in real life. More is accepted. So it is easier, in a way, to make contact with new people, or you can say other things, I feel, on social media (FG1-F2).

Conversely, online communications also had a downside, where communication was easily misinterpreted, as facial expressions, body language, and the tone of voice were largely missing. Beyond direct communication, actions or inactions on social media could be (mis)interpreted as social signals, and certain “norms” or rules of behaviour had to be followed. For example, failing to like and/or comment on a friend’s picture could be interpreted as a sign that something was wrong:

Yes, the pictures were nice, but you don’t always have to comment on every single one. But still, you feel like you need to, because…otherwise it may be like: “Oh, she didn’t comment on my picture!” It may be interpreted negatively (FG2-F3).

Some of the females discussed how they sometimes forgot to add someone to their private story, and worried that they had hurt someone unintentionally. Generally, both males and females agreed that the girls cared more and attached more meaning to social media than boys.

#### People behave worse on social media

The nature of social media was described as having some unfortunate effects on how people behave towards one another. On social media, people could be anonymous or feel anonymous, and according to the participants, it was easier to be mean because there were no real consequences of their behaviour, or because you did not see the emotional reaction of the other person:

That is perhaps the worst thing about social media, that people may be anonymous. They don’t feel that their behaviour have consequences for them, but… then they may post a nasty comment on a picture posted by a 13 year old girl and her self-esteem takes a hit. And then… They just think like “OK, it’s funny, I’ll just post a comment, she doesn’t know who I am.” And then it goes to hell for her (FG5-M1).

Negative and unwanted events on social media, actual or hypothesized, were thematised in all interviews. These events included backbiting, bullying, nasty comments, and threats to share content against one’s will.

Related to this, the girls in one interview expressed frustration about how the content people post on social media is freely discussed and criticized, where the social media profiles of celebrities and others are flooded with negative comments on their appearances: “It’s interesting that people bother to spend their time putting others down. Why would you, if you don’t care about that person, why would you spend your time commenting that you don’t think that person looks good in her picture?” (FG4-F2).

### Personal consequences of social media use

During the interviews, the participants discussed how social media use had impacted them personally in both positive and negative ways. Five subthemes were developed: (i) there are pros and cons of being connected, (ii) social hierarchies are on display, (iii) upward social comparison, (iv) the visibility and permanency of content, and (v) use on the expense of other things.

#### There are pros and cons of being connected

Social media was highly valued by the participants due to the possibility to effortlessly and continuously communicate with friends. Many highlighted that social media allowed them to stay in touch with friends and family living far away, making them “feel closer, even though they are on the other side of the world” (FG1-F2). In addition, social media provided a sense of connectedness with a wider community and gain insight into issues around the world.

Social media was further seen as positive in the sense that it allowed them to seek social support from friends, and that it was a means to express themselves and being heard/seen by others. Getting attention in terms of likes and comments, or being included in someone’s story, made them feel good. Conversely, the participants described how not being included in someone’s story or in a group chat made people feel excluded:

“I am in many different groups [on social media] with different groups of friends. And then, if one of my best mates see that I am in a group, and he’s sitting there and asks: “Hey, what is that? Why am I not in that group?” So, you can feel excluded” (FG3-M3)

The opportunity provided by social media to keep up with what their friends were doing was both valued and came with some unwanted consequences. It provided a feeling of “having control” of what was going on among their peers, but keeping up with everything happening on social media was also stressful. Several mentioned the constant stream of notifications as annoying and overwhelming: “Yes, you are often overwhelmed by everything. So it only leads to stress, more stress in your everyday life.” (FG2-F4).

Further, watching friends’ posts on social media of having fun without them, could make them feel unwanted or excluded. Also, it could trigger a feeling of missing out that drove them to attend social gatherings and parties despite having plans of relaxing at home, exemplified in the following dialogue:

FG1-F1: It’s that thing, that you feel that you should attend everything all the time.

FG1-F2: Yes, that you should join… Yes, I think social media contributes a lot to me feeling that I should do something every weekend.

FG1-M2: Yes.

FG1-F2: If it is a Friday or Saturday and I don’t have any plans, and it would have been wise and probably very cosy to relax at home with my family, and yeah, eat snacks and watch a movie. But I feel that I can’t. Because then I will just have a bad time, because I will see that everyone else is out having fun, and I’m just at home and… Still, they only post stuff when they are out. It seems like they are out every weekend, but it just that I have so many people on Snapchat. And when people share that they are out, it feels like everyone is.

#### Social hierarchies are on display

Another aspect of social media that impacted the participants negatively was the visibility of social relationships and social status on social media. Who were friends or not, who were allowed to see someone’s private story, etc., was readily visible and carried important meanings about the nature of peoples’ relationships:

And about private stories, I feel many people, sometimes they see like “my friend can see my other friend’s private story, but I can’t”. That does something to your mental health. And “why did she not add me to her story, she is included in my private story”, and it becomes like… (FG2-F5)

The participants described how the number of likes and comments people received and the number of friends/followers they had, quantified popularity and social status, which was tiresome to think about. Participants in all focus groups talked about themselves or others being preoccupied with comments and likes, where the lack of comments or likes triggered negative thoughts about how others perceived them.

#### Upward social comparison

The participants in all five groups talked about how comparing oneself to people on social media could trigger negative thoughts and feelings, and eventually lead to poor health. They described how people tended to portray themselves on social media in a positive way, thus creating a “positive bias” where everyone told a one-sided story of how successful and pretty they were and how many friends and fun experiences they had. They described how even though they knew that what was posted on social media was only one side of the story, it was hard to not compare one’s own life and appearance to it. This pertained both to peers and to celebrities. Some of the females suggested that it should be mandatory to tag pictures that had been digitally altered (“photo-shopped”), in order to reduce insecurity among young people.

But it’s not true. Every single one of those models have edited their photos. No one really looks like that. It is not true. Their skin is not that flawless, their bodies aren’t that perfect. And then you get ideas in your head that others are perfect and pretty, “I should look like that”. And then you get afraid and unsecure about yourself. Even though what you see is not real. But you choose to believe it. And that leads to poor health.” (FG4-F4).

Digitally altered images impacted not only peoples’ view of themselves, according to the females in one group, but also created unrealistic expectations among boys about what girls should look like. The widespread use of filters that augmented people’s facial features made some of the girls feel unsecure about their looks. They explained how the filters made them aware of their flaws and how much prettier they could have been if they looked more like how the filters made them look (plump lips, long lashes, etc.).

#### The visibility and permanency of content

The participants addressed some issues related to online communication or other online behaviours being visible and often permanent. Several participants talked about a specific feature of social media where the locations of your friends are visible in a map (*Snap Map*). Being visible on social media made them change their behaviour, or at least think about how their behaviour appeared on social media:

Since I have Snap Map, then I can think like; I am afraid that people might see that I am at home, almost. You think about it. “What if people see that I am at home all the time? Should I go to the gym, so that people see…?” You can almost start like that (FG1-F2).

On a similar note, the participants in three of the interviews described how easily photos could spread among peers and how people should be careful with sharing photos with others. Photos that they shared in confidence with one person could be distributed to other people without their consent or knowledge:

They don’t say it directly, that “I have a photo of you, I’m going to send it to that and that person.” But you know. You have it in the back of your mind when you think about it. “Oh, shit. I sent that photo. Now he or she has it. They may share it whenever they want to”, like (FG4-F3).

Further, the participants expressed worry that content they have posted never disappears and may harm them in the future. One participant stressed the necessity of using multiple user names so that what you do on social media would not be associated with you in real life.

#### Use on the expense of other things

Finally, many of the participants thought that they spent too much time on social media. The time and energy spent on social media displaced other important activities, such as doing homework or getting enough sleep:

It’s very addictive, yes, because it is a constant stream of entertainment and information, and I notice that it can have physical effects as well. Because I stay up when I should have gone to bed, because there’s so incredibly much to do and watch and keep yourself up to date on. And it’s fun, but you lose a lot of sleep (FG3-M1).

Several of the participants worried that their use of social media had some negative consequences for their personal development. Specifically, they talked about how they did not learn how to be bored or to get to know themselves because they always turned to social media when they had nothing to do: “I think that it’s very positive for your mental health to explore your thoughts and figure out who you are and stuff like that. And that is something that you lose [because of social media].” (FG1-F5) They described how they did not challenge themselves because they used social media as a sanctuary or protection if they found themselves in challenging social situations (something was awkward, they were in a room with strangers). For example, some mentioned that they picked up their phone instead of keeping conversations going and that social media use impaired their social skills.

### Motivations affecting social media use

Despite the negative effects discussed by the participants, they seemed highly motivated to use social media. Consequently, the participants gave the impression of being torn between the desire and perceived necessity to use social media and the negative consequences they experienced from it. Five subthemes were generated: (i) an unmissable social arena, (ii) self-presentation and impression management, (iii) from fun to addiction, (iv) a way to dodge what is difficult, and (v) awareness and regulation of own use.

#### An unmissable social arena

From the interviews, it was clear that one of the main motivations for using social media was to stay connected to and socialize with friends. As social media constituted such an important social arena, many of the participants expressed a necessity of using social media to keep up socially. Several participants described a fear of, and experiences with, missing out on what was happening among friends on social media. The participants stressed that there was no split between social media and real life and that if they missed out on things happening on social media, then they had missed “half the conversation”:

I think I would have become very stressed [without social media]. And that is because I think I’m afraid of the feeling of being left out. I would have felt like… it’s like if your friends went to hang out and you were left behind (FG1-F2).

In addition to this drive or desire to use social media, the participants described peer pressure to reply and provide likes and comments on friends’ content, or to reciprocate messages they received (i.e., streaks):

If my friend has posted a picture of herself, I feel that I have to comment on it, just because… it’s mandatory. But it’s not. I don’t know, it’s weird. It’s just the way it is, a culture that has evolved… (FG1-F1).

#### Self-presentation and impression management

A second important motivation for using social media was self-presentation. On social media, they could decide how they wanted others to perceive them, and their self-presentation was partly guided by social norms of what was considered cool among peers and showing others that they had an exciting life. Some of the females described it as a competition, where they deliberately posted pictures at times when people were most active, to get more feedback (likes, comments). Although the feedback they received on social media could produce positive feelings, they regarded it as unhealthy to care too much about it. Many expressed a desire to care less, but that it was hard to resist. As one participant put it: “But, it takes, I feel, quite a lot, psychologically, to resist the Instagram-pressure. You, sort of, you want to have a nice feed, you want many likes, and of course you want a lot of comments.” (FG2-F1) Others expressed that they rarely posted pictures on social media and that they cared little about how many likes or comments they received.

#### From fun to addiction

Most of the participants described social media as a source of entertainment and fun, and some described how they found creative inspiration on social media. In addition, they talked about their phone and social media as an easy escape from boredom, and that they had a habit of picking up their phone when they had nothing to do.

Many also described themselves and their peers as being addicted to social media. They described some features of their social media behaviour that resembled addictive behaviour, for example that there was something satisfying about using social media, that time went fast while using social media, and that they often spent more time than they intended: “Only you can decide when to stop [scrolling on social media], but not quite. It’s kind of your brain that decides and it becomes an addiction, or, it is natural to become addicted.” Others, in contrast, had no trouble leaving their phone behind and forgetting about social media for hours.

#### A way to dodge what is difficult

The participants discussed how they actively used their phone/social media to distract themselves from negative thoughts and feelings, and to escape awkward or uncomfortable social situations. It was also easier to talk about difficult things through social media because it became more distanced and less personal, and they could also take time to think about what they wanted to reply:

I think many people feel that it is easier to send a text message rather than saying things out loud, face-to-face. That it is easier to send it over the phone, because then the person receiving the text message can’t see how you react (FG4-F2).

#### Awareness and regulation of own use

The participants expressed a high level of awareness and reflection about their own use of social media. The participants described how they were less socially active on social media if they were having a bad day, and that they then would use social media mostly for entertainment. On a good day, some would be more socially active on social media, while others said they spent less time on social media when they are having fun.

I don’t know, if I’m having a good day then I usually don’t use my phone at all. Then I’m in such a good mood that I don’t need attention from anyone. I just sit and have a good time and just watch a really good movie and eat junk food. Just be pleased with that day, sort of. And then I forget my phone, forget social media, forget Snapchat, and just don’t answer and people will have to call me (FG4-F4).

One participant explained how he could be more vulnerable to social comparison if he had a bad day.

The participants talked about how they had become more relaxed about social media as they aged. At a younger age, they were more affected in terms of body image, social comparison, or negative comments, and had grown more robust based on their own experiences.

I have put it behind me now. Because I’m thinking, does it matter? They’re not real. But before it was like: “Oh, they’re so pretty and skinny, blah, blah.” And then I let myself be affected and my health was affected, or I put myself down (FG4-F4).

Several expressed the importance of being able to put away their phone, for example when doing homework. Many of the participants expressed a desire to use social media less, and several told about positive experiences while not having access to social media, typically when on vacation:

FG2-F1: Then it was nice to have, like, a week of relaxation. Like a… yes, cell phone rehab.

Moderator: What was nice about that, not having…

FG2-F1: Well, you didn’t have to write, like write text messages to anyone or, you sort of had an excuse not to be online. If that makes sense. You relax more, like, I was much more relaxed, and like, I could, rather than spending time on Instagram, I could talk to my family. Something that happens rarely in my everyday life. I enjoyed that very much.

Many of the participants took specific actions to regulate their use of social media, such as muting their phone and disabling notifications. The participants stressed how the algorithms of social media were designed to encourage more use.

In three of the interviews, the participants expressed the need for more relevant information about the negative effects of social media. They pointed out that children and adolescents need rules regarding social media use, and that when they start using social media, it is hard to stop. Conversely, some of the participants thought that grown-ups had a very negative view of social media, and that social media use was seen as more problematic than it was:

FG5-M3: There is a lot of positive sides to social media as well. But teachers and parents often bring up the negative sides. “You spend too much time on it” and “can you please put it away now?” and “social media is negative for your mental health”. That is what we are told by parents and other grown-ups.

Moderator: What do you think of that?

FG5-M3: It’s a bit annoying. Many of them haven’t really familiarized themselves with social media, they don’t know what we do in our world, on social media and stuff.

Similarly, the males in one of the focus groups talked about how parents were disinterested in gaming and how they wished their parents were more engaged.

## Discussion

**Fig. 1:**
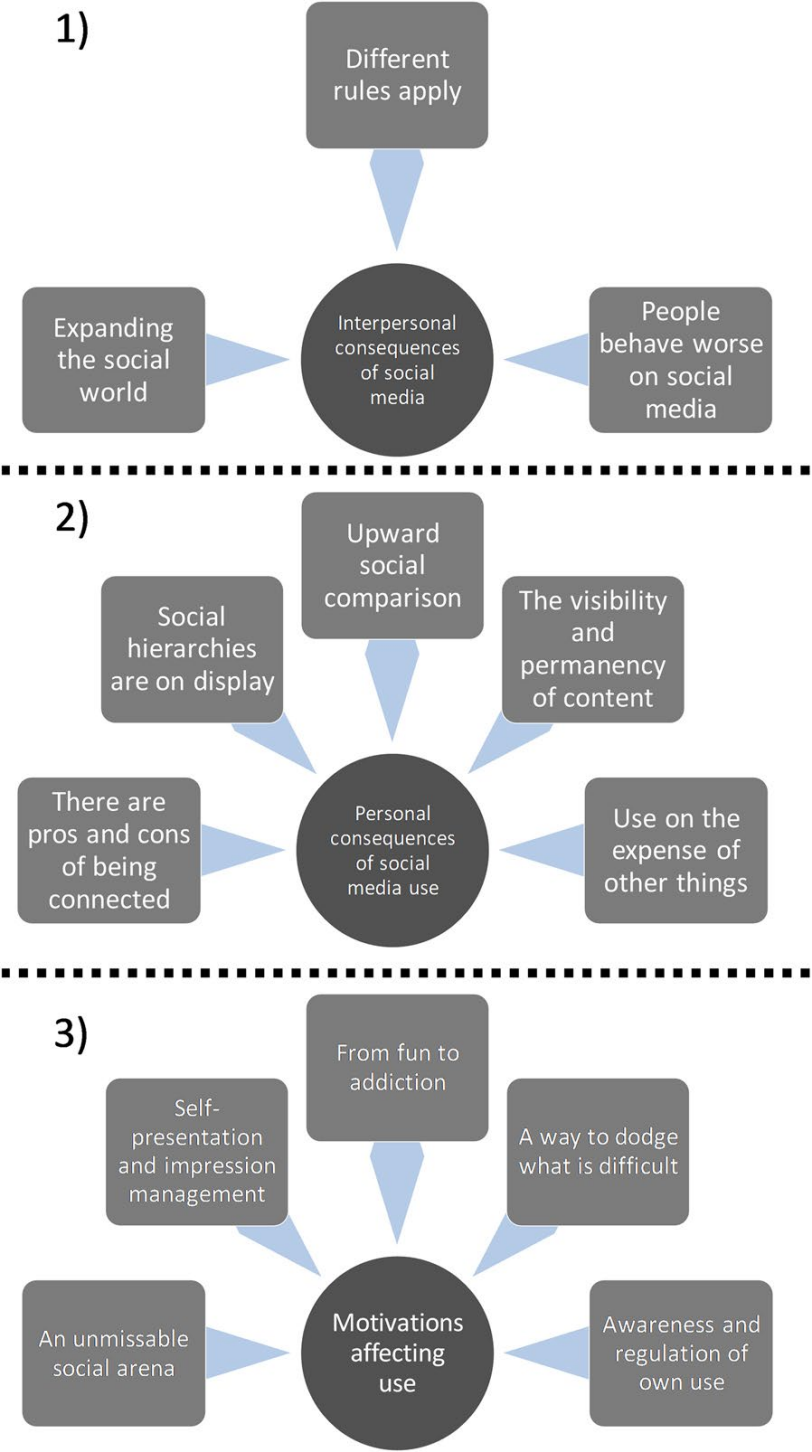
A schematic overview of the themes (circles) and subthemes (squares). (1) Interpersonal consequences of social media, (2) personal consequences of social media use, and (3) motivations affecting use

In this study, 27 adolescents gave their perspectives on the relationship between social media use and mental health. Three main themes were developed, offering an in-depth account of the issues that were discussed in the focus group interviews. Figure 1 provides a schematic overview of the themes and subthemes. Overall, it was clear that the participants saw social media as a positive and necessary aspect of their lives, while also recognizing several ways in which social media could negatively influence their well-being. This Janus-faced characteristic of social media is in line with previous qualitative findings [24, 25, 27]. The discussion among the participants revealed that, from their perspective, social media use catalyses changes to how people communicate and relate to each other (theme 1). Some changes were positive, in terms of facilitating social connectedness, while others were negative, such as people behaving worse on social media than in face-to-face interaction. Further, social media affected the participants at the individual level (theme 2); positively in terms of a sense of belonging and social support, and negatively in terms of perceived stress, body pressure, and displacing other activities such as sleep and homework. The motivations for using, or not using, social media were multifaceted (theme 3), reflecting the positive and negative aspects of social media in terms of interpersonal and personal consequences.

### The dark side of social media

The present findings show that adolescents consider social media to have a range of negative influences on their mental health and well-being. In line with another qualitative study [26], the participants highlighted appearance-related social comparison as one mechanism through which social media may lead to poor mental health, captured under the theme “Personal consequences of social media use”. As outlined by Firth et al. [37], upward social comparison is an implicit cognitive process, which may become harmful when active on social media, where people are frequently exposed to highly successful individuals. This resonates well with the narratives of the participants in this study: That even though they knew that social media only show one side of the story, they struggled not to compare themselves to content they are exposed to. Some of the participants suggested that all photo-shopped images (digitally edited to improve people’s physical appearance) should be tagged, to reduce insecurity among young people. The participants in the study by O’Reilly et al. [26] similarly identified photo-shopped images on social media as a cause of low self-esteem among adolescents. Studies have shown how media exposure to “body perfect” ideals can lead to body dissatisfaction [38–40], increasing the risk of low self-esteem and depression [41]. On a similar note, a few of the female participants discussed the widespread use of filters that augment people’s facial features, and how these filters made them aware about how their own features could be improved. This may represent an even more powerful influence than comparing oneself to other people. Recently, the term “Snapchat dysmorphia” was coined, following interviews with plastic surgeons reporting how patients requested procedures to make them look like their “filtered self” [42]. The present study hints that filter use on social media may contribute to insecurities about one’s appearance, particularly among girls.

Anonymity is a well-established facilitator of negative online behaviour [43, 44], which was captured in the theme “Interpersonal consequences of social media”. The participants described how negative behaviour on social media was facilitated by not seeing the emotional reaction of the recipient. This is in line with the notion of reduced ‘social presence’ in online communication, i.e., a reduction of non-verbal cues, making it easier to engage in deliberate negative online behaviours [45]. The negative events on social media discussed by the participants, such as backbiting, negative comments, and bullying, have also been identified in quantitative studies. For example, one third of European 15–16 year-olds have reported having negative experiences online that made them feel scared, upset, or uncomfortable [46].

According to Marwick and Boyd [47], adolescents attempt to achieve privacy online using strategies to control what information about them is consumed by whom. This resonates well with our subtheme “The visibility and permanency of content”, where several participants mentioned the permanency of online content as a down-side of social media. Several expressed frustration that content they had posted years ago still appeared if someone “googled” them, and were careful about what they posted online in fear of future consequences, for instance in relation to career prospects. One of the participants reported having several social media accounts under pseudonyms, to avoid that content was linked to him in real life. These narratives may be paralleled with the desire to “*be* in public without always *being* public” reported in other research [47, p. 1052]. Recent developments of social media platforms allow users to effortlessly select who may see which content, either by hiding content from specific people or selecting a group of people that are permitted to see their content (e.g., private stories on Snapchat) [48]. Although such developments may be regarded as a positive feature in terms of navigating privacy, the participants in the present study highlighted how not being included in someone’s private story could make them feel left out, as captured in the subtheme “Social hierarchies are on display”.

### Staying connected

Despite recognizing a range of ways social media affected them negatively, the participants were highly motivated to use them. Our subtheme “An unmissable social arena” captures the importance of social media as an arena for peer interaction, which arguably is the main reason adolescents use social media. The need to be a part of the online community has also been found in other qualitative studies [24, 27]. These findings resonates well with the dual-factor model presented by Nadkarni and Hofman [49] stating that alongside self-presentation, the need to belong is the main motivational force driving social media use. As captured in the subtheme “There are pros and cons of being connected”, the opportunities provided by social media to stay continuously in touch with friends and family was highly valued. Social media was regarded as a source of social support, and the participants likened their cell phone to having their friends in their pocket, instantly available when needed. This was also thematised in the qualitative study by Weinstein [25], where the participants expressed that having their friends accessible caused a sense of connection or sense of having people with them. Strong peer relationships and having a support network are crucial to well-being [50, 51], and the opportunities provided by social media to stay connected with others may thus be considered a major positive contribution to mental health. In line with this, quantitative research has found that Facebook use promotes subjective well-being and social capital [52–54]. Beyond such positive relational aspects, experiences such as a fear of missing out (FoMo), captured in the subtheme “An unmissable social arena”, also seems to be a major contributor to social media use. FoMo has been defined as “a pervasive apprehension that others might be having rewarding experiences from which one is absent”, and further that “FoMo is characterized by the desire to stay continually connected with what others are doing” [55, p. 1841]. Although the emotional component of FoMo is probably an inherent part of being human, and particularly among adolescents, it is likely intensified by social media, by amplifying the behavioural part of FoMo, i.e., staying connected with what others are doing. In addition, expectations of reciprocity seem to encourage social media use. For example, not responding or providing appropriate feedback on friends’ content could be interpreted as expressing negative feeling towards them, and thus certain actions had to be carried out in order to avoid causing “drama”. This was particularly a concern among the female participants. Such fear of being impolite or cause “drama”, has also been found in other qualitative studies [24, 25].

### Striking a balance

One important finding was the desire to use social media less, captured under the theme “Motivations affecting social media use”. Several participants described periods without internet access as liberating. Not having internet access was perceived as an excuse to not respond on social media, escaping the expectation of reciprocity and constant availability. They did not, however, manage to maintain abstinence when they returned to their everyday life. In fact, many of the participants described themselves and their peers as addicted to social media, which has also emerged in other qualitative studies [26]. A few of the participants focused on how social media is developed to make them use it more. One likely culprit is “the filter bubble”, where the algorithms of social media platforms make endless recommendations for new content based on an individual’s previous behaviour on social media [56]. Interestingly, many of the participants in the present study seemed to blame themselves for not having the will-power to disengage from social media, not the design of the algorithms, as captured in the theme “Awareness and regulation of own use”. Importantly, this subtheme also reflects the participants’ efforts to minimize the negative effects of social media by for example disabling notifications and putting the phone in silence mode during school hours.

### Implications

The present results highlight that adolescents depend on social media as a source of peer interaction. Thus, any interventions or guidelines regarding social media use among adolescents need to take this into consideration in order to avoid hampering their social lives.

Conversely, the participants discussed a range of negative consequences of social media, and expressed a desire to reduce their social media use. Thus, social media should not plainly be accepted as something that “is here to stay”. Rather, users should actively consider how they want to utilize the positive aspects of social media and limit the negative consequences. And in theory, it is fully possible to regulate the level of engagement with social media. From this study, however, it was clear that adolescents are highly motivated to use social media despite recognizing that social media have several negative effects on them. Thus, protecting children and adolescents from the potential harmful effects of social media, while utilizing the positive aspects, should be a task for parents, teachers, policymakers, and scientists now and in the future. Responsibility also lie with the providers of social media platforms, and some have highlighted providers’ unwillingness or inability to effectively account for children’s heightened vulnerability on social media [57, 58]. In the UK, several proposals are considered to compel social media providers into taking more concerted action, most of them adapting a legally-driven and law-enforcement based approach as opposed to a self-regulatory approach [57]. Because of their relatively limited capacity for self-regulation, this approach might prove to be more fruitful than focusing interventions on children and young people, as social media is designed to promote as much use as possible [59].

Adolescents now face some specific challenges that were not faced by previous generations. Specifically, as much of their social lives and their self-expression happen online, their behaviour may become permanent and retrievable, which may cause worry and embarrassment. Another challenge is to be productive and focused while having access to their mobile phones [60, 61], which competes for their attention through offering access to peer interaction and endless entertainment. No other generation have had to restrain themselves in the face of such powerful distractions. These challenges, and how they affect mental health, need to be taken into considerations in endeavours to improve mental health among adolescents.

### Strengths and limitations

The results of the present study should be interpreted in light of the specific context of Norwegian adolescents. With this limitation in mind, the participants can be argued to represent a diverse subset of adolescents as they attended two different schools (urban and rural settings) and different educational programs, including general studies, vocational training programs, and an e-sport program. Considering the widespread use of social media among adolescents, the participants may be considered highly competent on the subject of interest.

Qualitative research can contribute to develop knowledge and lead us to think differently about subjects where knowledge is weak or lacking [28]. Focus groups have the strength that they introduce group dynamics that may contribute to mobilize associations and create stories [28]. Conversely, using a focus group methodology may have elicited unwanted group dynamics. In a focus group setting, one or a few participants can dominate the discussion and influence which opinions are regarded popular or relevant [28], and we may therefore have lost relevant perspectives from less outgoing and assertive adolescents. Researchers may need to consider collecting data based on individual interviews in order to capture more divergent views among participants.

Another important limitation is that this study is based on self-reported experiences, and future studies also need to investigate what adolescents actually do on social media, and how this behaviour is related to mental health and well-being. This may for example be achieved by sampling moment-to-moment behaviour and emotional states.

It is important to consider how the background of the analytic team might have influenced the results, i.e., the interpretative validity of the study [62]. The research group was quite homogenous, as four out of five authors (GJH, VS, MV, and JCS) are trained clinical psychologists based on the scientist-practitioner model. Consequently, the experiences and motivations of the individual may have been placed on centre stage to a larger degree than if the material was analysed by researchers from other backgrounds. Furthermore, none of the co-authors can be considered to be digital natives, and the analytic process could have benefitted from including someone who had in-depth experiences with social media.

## Conclusion

In conclusion, this study shows that the adolescents had a range of negative perceptions about, attitudes towards, and experiences with social media. At the same time, they were highly motivated to use social media by aspects they experienced as positive. The adolescents struggled to balance social media and real life, expressing a desire to be less preoccupied with social media. Considering the compelling nature of social media and adolescents’ relatively limited self-regulatory capacities, efforts to regulate social media use should avoid relying on self-regulation, and rather draw on parents, teachers, policy makers, and social media providers, while also recognizing the importance of social media as an arena for peer interaction. The present study shows that social media use is multifaceted, driven by a range of motivational factors, and is highly varied across individuals, and thus that broad generalizations about social media are unlikely to be helpful. The results also hints about gender differences. The findings may inform future studies and provide adolescent’s perspectives into interventions and policies.

## Supplementary Information

**Additional file 1:** The interview guide

## Data Availability

The transcripts of the interviews are not publicly available due to privacy related issues. Information on transcripts is available from the corresponding author on reasonable request.
